# A novel comparative study of NNAR approach with linear stochastic time series models in predicting tennis player's performance

**DOI:** 10.1186/s13102-024-00815-7

**Published:** 2024-01-25

**Authors:** Abdullah M. Almarashi, Muhammad Daniyal, Farrukh Jamal

**Affiliations:** 1 Department of Statistics, Faculty of Science, King Abdulaziz University, 21589 Jeddah, Saudi Arabia; 2 Department of Statistics, Faculty of Computing, The Islamia University of Bahawalpur, Punjab, 63100 Pakistan

**Keywords:** Forecasting, Neural Networking, NNAR, Nonlinear time series, TBATS, Modelling

## Abstract

**Background:**

Prediction models have gained immense importance in various fields for decision-making purposes. In the context of tennis, relying solely on the probability of winning a single match may not be sufficient for predicting a player's future performance or ranking. The performance of a tennis player is influenced by the timing of their matches throughout the year, necessitating the incorporation of time as a crucial factor. This study aims to focus on prediction models for performance indicators that can assist both tennis players and sports analysts in forecasting player standings in future matches.

**Methodology:**

To predict player performance, this study employs a dynamic technique that analyzes the structure of performance using both linear and nonlinear time series models. A novel approach has been taken, comparing the performance of the non-linear Neural Network Auto-Regressive (NNAR) model with conventional stochastic linear and nonlinear models such as Auto-Regressive Integrated Moving Average (ARIMA), Exponential Smoothing (ETS), and TBATS (Trigonometric Seasonal Decomposition Time Series).

**Results:**

The study finds that the NNAR model outperforms all other competing models based on lower values of Root Mean Squared Error (RMSE), Mean Absolute Error (MAE), and Mean Absolute Percentage Error (MAPE). This superiority in performance metrics suggests that the NNAR model is the most appropriate approach for predicting player performance in tennis. Additionally, the prediction results obtained from the NNAR model demonstrate narrow 95% Confidence Intervals, indicating higher accuracy and reliability in the forecasts.

**Conclusion:**

In conclusion, this study highlights the significance of incorporating time as a factor when predicting player performance in tennis. It emphasizes the potential benefits of using the NNAR model for forecasting future player standings in matches. The findings suggest that the NNAR model is a recommended approach compared to conventional models like ARIMA, ETS, and TBATS. By considering time as a crucial factor and employing the NNAR model, both tennis players and sports analysts can make more accurate predictions about player performance.

## Introduction

Sports performance prediction has significant implications for the development of scientific training methods that align with evolving trends in sports performance [1]. This enables athletes, coaches, schools, sports teams, and sports training institutions to reform physical education and training based on informed opinions. Accurately predicting sports achievement plays a vital role in improving sports training and teaching by uncovering regular factors and characteristics of human training [2]. Therefore, the prediction of sports performance has been a prominent topic in sports research. However, accurately predicting a player's performance using traditional methods is challenging due to the complex interactions among various influencing factors [3]. Consequently, studying sports performance prediction models holds great significance in promoting scientific training and enhancing sports performance [4]. Sports, being a highly attractive activity in modern society, exert a profound and extensive influence on the development of sports culture, which in turn impacts other related cultures [5]. Deep neural networks (DNN) have been proposed as an alternative method for predicting sports performance, directly impacting training and preparation goals and facilitating the discovery of performance development rules [6–8]. The complexity of predicting sports performance arises from the numerous variables involved, including changes in human characteristics, age, and environmental factors [3]. Building sports performance prediction models requires multivariate and multi-parameter statistical analysis, incorporating topics such as statistics, information processing, and modern mathematics. Selecting an appropriate and highly precise method is crucial for successful forecasting.

Time series models have gained attention in various fields of life, including sports such as soccer, golf, cricket, and tennis, where accurate predictions of sports results have always fascinated the sporting world [9–13]. Multimedia, social media, and television provide insightful coverage of sporting tournaments through predictions [14]. In recent years, predicting and modeling tennis match results, in particular, have received significant consideration [15, 16]. Experts have utilized various predictors in classification algorithms to forecast tennis match outcomes, including the application of the Bradley Terry-type model in predicting outcomes for the top men's professional ATP tour and the use of high-dimensional models [17, 18]. Furthermore, experiments have explored the feasibility of modeling to forecast soccer players' readiness to play and reduce sports injuries. A study focused on predicting readiness to play by utilizing a Long Short-Term Memory Recurrent Neural Network (LSTM RNN) based on a dataset from two male high-division soccer teams in Norway. The study demonstrated the value of this approach in predicting the reported training load, including positive and negative peaks [19].

Traditional methods of predicting sports performance suffer from drawbacks such as high computational costs and poor adaptive anti-interference of parameters, leading to low prediction accuracy. However, deep neural studies offer stable effectiveness, adaptability, and the ability to determine linear correspondences in uncertain input–output function mapping, making them widely used in various fields [20]. A study [21] established that neural network models provide more accurate predictions of sports performance and better evaluation of physical quality development compared to traditional methods. This model brings great convenience to sports performance prediction, enhancing modeling efficiency and prediction accuracy. Previous studies have utilized conventional statistical models to describe key features of tennis matches and assess players' abilities in various scenarios [22, 23]. These models have proven effective in constructing rankings, determining entry and seeding in tennis tournaments, providing match and tournament predictions, and testing the efficiency of betting markets [16]. The Bradley-Terry model is commonly used for statistical analysis of tennis matches [24]. Moreover, ATP rankings points have been employed to gauge the level of strength among tennis players [9, 25].

However, traditional methods for predicting sports outcomes overlook the presence of linear and non-linear patterns in players' performance, resulting in low prediction accuracy. In contrast, modern machine learning techniques such as NNAR incorporate both linear and non-linear patterns, making them more widely adopted. Hence, this study aims to establish a sports performance prediction model for tennis players using NNAR and analyze its reliability by comparing it with conventional linear time series techniques. The findings demonstrate that the NNAR-based performance prediction model outperforms traditional prediction methods in accurately forecasting performance indicators. This model can significantly enhance the convenience of sports performance prediction and further improve modeling efficiency and prediction accuracy.

## Data and Methods

The data used to model the performance of three tennis players was sourced from (https://www.ultimatetennisstatistics.com/playerProfile?playerId=4742&tab=timeline). For each player, the data range from 2004 to 2022 was selected consisting of twenty sample points or size for each player and for each indicator. The primary performance indicators chosen for each player included their probability of winning, number of ACES, game dominance, and double faults per year. These performance indicators were utilized as training datasets for modeling the neural network models. To compare the performance of conventional time series models and neural auto-regressive (NNAR) models, the key performance indicators were examined. The main assumption underlying these models was that the players would continue playing throughout the forecasted period of five years.

## Methods for modelling and forecasting

The methodology used for time series modeling and forecasting involves obtaining meaningful statistical measures and characteristics of the time series data. The ARIMA model can be represented as ARIMA (**p**,**d**,**q**), where p represents the order of the autoregressive component, d indicates the differenced trend, and q signifies the order of the moving average component. The equations representing the AR (p) and MA (q) time series models are as follows;

1 $${{\varvec{Y}}}_{{\varvec{t}}}=\boldsymbol{ }{\boldsymbol{\varphi }}_{1}{{\varvec{Y}}}_{{\varvec{t}}-1}+\boldsymbol{ }{\boldsymbol{\varphi }}_{2}{{\varvec{Y}}}_{{\varvec{t}}-2}+\dots +\boldsymbol{ }{\boldsymbol{\varphi }}_{{\varvec{p}}}{{\varvec{Y}}}_{{\varvec{t}}-{\varvec{p}}}+{{\varvec{\upvarepsilon}}}_{{\varvec{t}}}$$

2 $${{\varvec{Y}}}_{{\varvec{t}}}=\boldsymbol{ }{{\varvec{\theta}}}_{1}{{\varvec{\varepsilon}}}_{{\varvec{t}}-1}-\boldsymbol{ }{{\varvec{\theta}}}_{2}{{\varvec{\varepsilon}}}_{{\varvec{t}}-2}-\dots -\boldsymbol{ }{{\varvec{\theta}}}_{{\varvec{q}}}{{\varvec{\varepsilon}}}_{{\varvec{t}}-{\varvec{q}}}+{{\varvec{\varepsilon}}}_{{\varvec{t}}}$$

where ${{\text{Y}}}_{{\text{t}}}$ is observed or output value of time series,$\mathrm{\varphi },$ and $\uptheta$ are the coefficients of AR and MA models respectively and ${\upvarepsilon }_{{\text{t}}}$ shows the residual value at time ${\text{t}}$. The generalized form of ARMA model has the following expression;

3 $${{\varvec{Y}}}_{{\varvec{t}}}=\boldsymbol{ }\boldsymbol{\alpha }+{\boldsymbol{\varphi }}_{1}{{\varvec{Y}}}_{{\varvec{t}}-1}+\boldsymbol{ }{\boldsymbol{\varphi }}_{2}{{\varvec{Y}}}_{{\varvec{t}}-2}+\dots +\boldsymbol{ }{\boldsymbol{\varphi }}_{{\varvec{p}}}{{\varvec{Y}}}_{{\varvec{t}}-{\varvec{p}}}+{{\varvec{\upvarepsilon}}}_{{\varvec{t}}}-\boldsymbol{ }{{\varvec{\theta}}}_{1}{{\varvec{\varepsilon}}}_{{\varvec{t}}-1}-\boldsymbol{ }{{\varvec{\theta}}}_{2}{{\varvec{\varepsilon}}}_{{\varvec{t}}-2}-\dots -\boldsymbol{ }{{\varvec{\theta}}}_{{\varvec{q}}}{{\varvec{\varepsilon}}}_{{\varvec{t}}-{\varvec{q}}}$$

where $\mathrm{\alpha }$ shows the constant term, and ${\upvarepsilon }_{{\text{t}}-1}$ is the past residual noise term. The ARMA model can be converted into the ARIMA model which deals with the non-stationary time series. The non-stationary time series can be made stationary by differencing.

## The modeling methodology has the following steps:

**Fig. 1:**
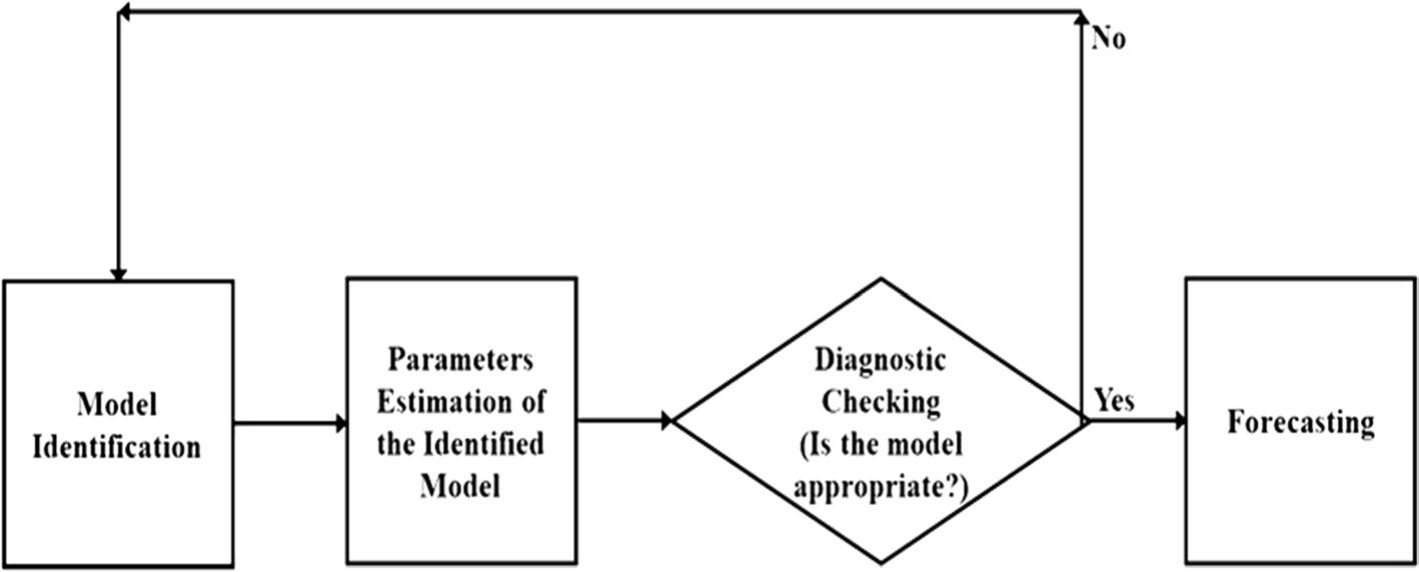
Flowchart of steps involved in the modelling

1. *Identification*: The model identification process necessitates that the time series exhibits stationarity, and that the model parameters remain independent of time. Frequently, the time series does not possess the characteristics of white noise initially, thus requiring differencing to transform it into a similar pattern. To ascertain the stationarity of the series, we employ a statistical test known as the Augmented Dickey-Fuller (ADF) test, which assumes the null hypothesis that the series is non-stationary. Once we have achieved stationarity in the series, we then employ graphical tools like the autocorrelation function (ACF) and partial autocorrelation function (PACF) to ascertain the appropriate order of the candidate model.
2. *Estimation*: In the model estimation phase, we visually analyze the autocorrelation function (ACF) and partial autocorrelation function (PACF) of the series in order to estimate the suitable candidate model for the dataset. Various combinations of candidate models are tested, and the final model is chosen based on accuracy parameters criteria.
3. *Diagnostics***:** In the diagnostic checks, the selected candidate model undergoes evaluation using diagnostic tools such as mean absolute error (MAE), root mean square error (RMSE), and mean absolute percentage error (MAPE). The model that achieves the lowest values for these metrics is considered one of the best models for subsequent steps.
4. *Forecasting*: In the forecasting step, we utilize the candidate model that satisfies all the aforementioned conditions to predict future values of the data series. Figure 1 shows the flowchart of all four steps explained for modeling the ARIMA model.

### TBATS (Trigonometric Seasonal Decomposition Time Series)

TBATS is a nonlinear time series model which handles the data series having several seasonal patterns, i.e., the pattern of the data changes its behavior over time. The Trigonometric seasonality (TBATS) method is favored over BATS due to its ability to handle intricate and high-frequency patterns. The TBATS model can be expressed as follow;

4 $$\begin{array}{cc}\begin{array}{l}{{\varvec{x}}}_{{\varvec{t}}}^{(\boldsymbol{\alpha })}={{\varvec{\tau}}}_{{\varvec{t}}-1}+{\varvec{\beta}}{{\varvec{k}}}_{{\varvec{t}}-1}+\sum_{{\varvec{i}}=1}^{{\varvec{T}}}{\boldsymbol{\alpha }}_{{\varvec{t}}-{{\varvec{n}}}_{{\varvec{i}}}}^{({\varvec{i}})}+{\boldsymbol{\vartheta }}_{{\varvec{t}}}\\ {{\varvec{\tau}}}_{{\varvec{t}}}={{\varvec{\tau}}}_{{\varvec{t}}-1}+{\varvec{\beta}}{{\varvec{k}}}_{{\varvec{t}}-1}+{\varvec{\gamma}}{\boldsymbol{\vartheta }}_{{\varvec{t}}}\end{array}\\ {{\varvec{k}}}_{{\varvec{t}}}=\boldsymbol{ }{\varvec{\beta}}{{\varvec{k}}}_{{\varvec{t}}-1}+{\varvec{\omega}}{\boldsymbol{\vartheta }}_{{\varvec{t}}}\\ {\boldsymbol{\vartheta }}_{{\varvec{t}}}=\sum_{{\varvec{i}}=1}^{{\varvec{p}}}{{\varvec{\delta}}}_{{\varvec{i}}}{\boldsymbol{\vartheta }}_{{\varvec{t}}-1}+\sum_{{\varvec{i}}=1}^{{\varvec{q}}}{{\varvec{\theta}}}_{{\varvec{i}}}{{\varvec{\varepsilon}}}_{{\varvec{t}}-1}+{{\varvec{\varepsilon}}}_{{\varvec{t}}}\end{array}$$

The seasonal component of the TBATS is given by;

5 $$\begin{array}{cc}{{\varvec{z}}}_{{\varvec{t}}}^{({\varvec{i}})}=\sum_{{\varvec{i}}=1}^{({{\varvec{l}}}_{{\varvec{i}}})}{{\varvec{z}}}_{{\varvec{j}},{\varvec{t}}}^{({\varvec{i}})}\\ {{\varvec{z}}}_{{\varvec{j}},{\varvec{t}}}^{({\varvec{i}})}={{\varvec{z}}}_{{\varvec{j}},{\varvec{t}}-1}^{({\varvec{i}})}{\varvec{cos}}\left({{\varvec{\xi}}}_{{\varvec{i}}}\right)+{{\varvec{z}}}_{{\varvec{j}},{\varvec{t}}-1}^{\boldsymbol{*}({\varvec{i}})}{\varvec{sin}}\left({{\varvec{\xi}}}_{{\varvec{i}}}\right)+{{\varvec{\phi}}}_{1}^{({\varvec{i}})}{\boldsymbol{\vartheta }}_{{\varvec{t}}}\\ {{\varvec{z}}}_{{\varvec{j}},{\varvec{t}}}^{\boldsymbol{*}({\varvec{i}})}={-{\varvec{z}}}_{{\varvec{j}},{\varvec{t}}-1}^{({\varvec{i}})}{\varvec{sin}}\left({{\varvec{\xi}}}_{{\varvec{i}}}\right)+{{\varvec{z}}}_{{\varvec{j}},{\varvec{t}}-1}^{\boldsymbol{*}({\varvec{i}})}{\varvec{cos}}\left({{\varvec{\xi}}}_{{\varvec{i}}}\right)+{{\varvec{\phi}}}_{2}^{({\varvec{i}})}{\boldsymbol{\vartheta }}_{{\varvec{t}}}\end{array}$$

where ${\upxi }_{{\text{i}}}=\frac{2\mathrm{\pi j}}{{{\text{n}}}_{{\text{i}}}}$ and ${\upphi }_{1}^{({\text{i}})}$, ${\upphi }_{2}^{({\text{i}})}$ are the seasonal smoothing.

### Exponential Smoothing (ETS)

Exponential smoothing (ETS) can be applied to data having both systematic trends and seasonal components. It is a significant forecasting methodology that can be applied as an alternative to ARIMA techniques. Models were evaluated by using the R-package “ets()” function.

### Neural Network Autoregressive Model (NNAR)

In modeling process, our focus was on the NNAR model as the machine learning model. We employed an automated selection method to determine the appropriate number of hidden layers. Systematically varying the number of hidden layers and neurons allowed us to obtain the most accurate models [26]. It is worth mentioning that neural networks lacking hidden units are essentially equivalent to linear statistical forecasting techniques [27]. Hidden units play a vital role in neural networks as they facilitate the mapping between input and output variables, while also introducing nonlinearity. Moreover, they aid in identifying patterns within the dataset [26]. In the context of time-series data, lagged values can be utilized as input data for a neural network, similar to how they are employed in a linear autoregressive model.

An NNAR ($p$,$kp,k$) shows that hidden layer has $pp$ delayed inputs and $kk$ nodes. Moreover, NNAR ($p$,$0p,0$) model is the same as an ARIMA ($p$,$0p,0$), but without parameter limitations that assure stationarity. The expression is constructed in two stages. The $K$ activations come first. In the activation, $A\left(k\right), k=1, \dots ,K$, the hidden layer is calculated as a function of the input characteristics ${X}_{j}={X}_{t-1,}\dots , {X}_{t-p,}$ with

6 $${\varvec{A}}\left({\varvec{k}}\right)={\varvec{h}}\left({\varvec{k}}\right)={\varvec{g}}\left({{\varvec{w}}}_{{\varvec{k}}0}+\sum_{{\varvec{j}}=1}^{{\varvec{p}}}{{\varvec{w}}}_{{\varvec{k}}{\varvec{j}}}{{\varvec{X}}}_{{\varvec{j}}}\right)$$

where $g$ is a previously defined nonlinear activation function. Each $A\left(k\right)$ maybe seen as a separate ${h}_{k}\left(X\right)$ transformation of the unique characteristics. The output layer receives these $K$ instigations from the hidden layer

7 $${\varvec{f}}\left({\varvec{X}}\right)={{\varvec{\beta}}}_{0}+\sum_{{\varvec{k}}=1}^{{\varvec{K}}}{{\varvec{\beta}}}_{{\varvec{k}}}{\varvec{A}}\left({\varvec{k}}\right)$$

In NNAR modeling, the sigmoid activation function, which is identical to the logistic regression function, is employed. This activation function serves the purpose of transforming a linear function into a probability ranging from 0 to 1. The sigmoid activation function can be represented by the following mathematical form as follow.

8 $$\mathbf{g}\left(\mathbf{z}\right)=\frac{\mathbf{e}\mathbf{x}\mathbf{p}(\mathbf{z})}{1+\mathbf{e}\mathbf{x}\mathbf{p}(\mathbf{z})}=\frac{1}{1+\mathbf{e}\mathbf{x}\mathbf{p}(-\mathbf{z})}$$

**Fig. 2:**
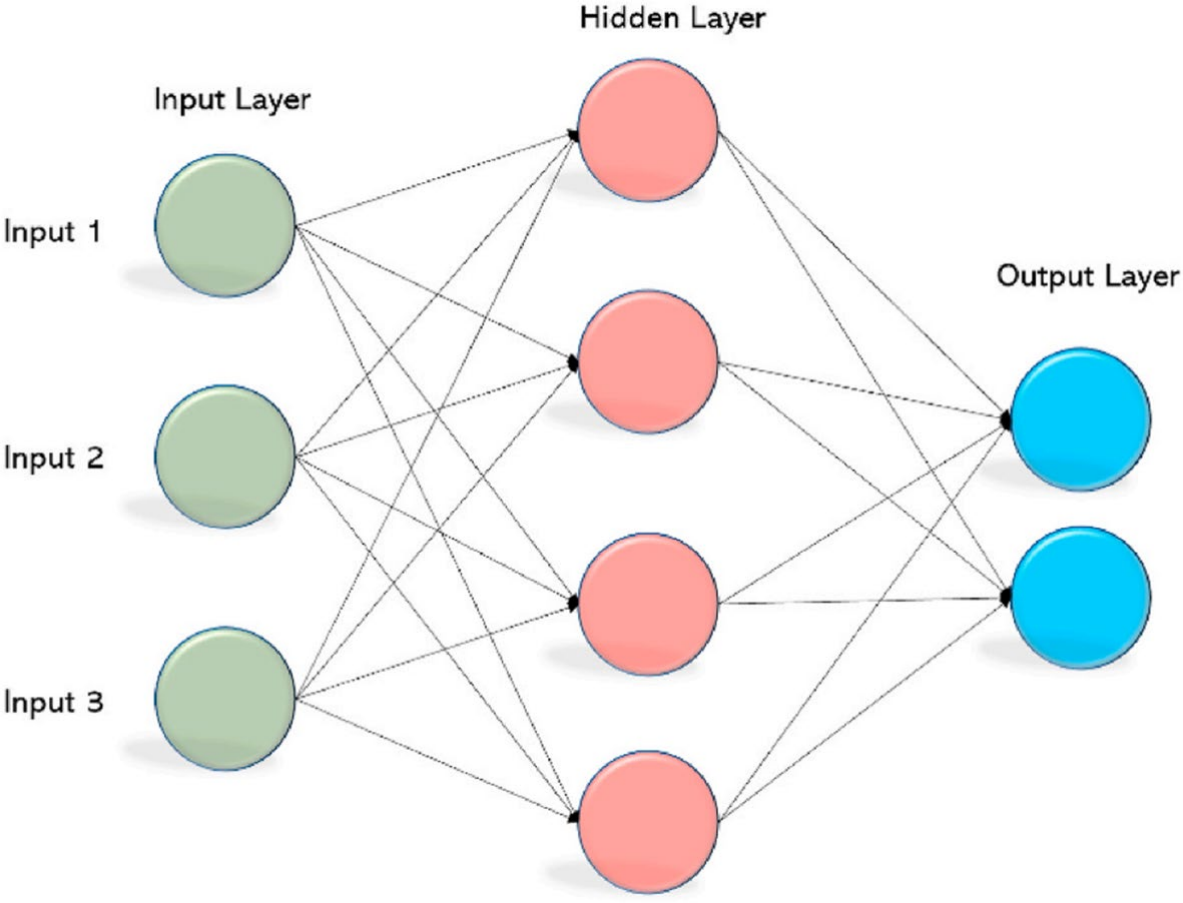
NNAR structure with input, hidden, and output layers

Figure 2 shows the structure of NNAR model with input, hidden, and output layers.

For analyzing the performance of all forecasting models, MAE, RMSE, and MAPE as the model selection criteria. The best model will be chosen utilizing the RMSE, MAE, and MAPE criteria and forecasting. MAE stands for Mean Absolute Error. It is a metric used to measure the average difference between the predicted and actual values in a model. MAE is often used as a performance metric in machine learning and statistical modeling tasks. It provides a measure of the average magnitude of errors made by a model, regardless of their direction (positive or negative). The lower the MAE value, the better the model's performance in terms of prediction accuracy. RMSE stands for Root Mean Square Error. It is another metric commonly used to evaluate the performance of a regression model. RMSE measures the average magnitude of the differences between predicted and actual values, similar to MAE, but it gives more weight to large errors due to the squaring operation. MAPE stands for Mean Absolute Percentage Error. It is a metric used to assess the accuracy of a forecasting model, particularly in the context of time series analysis and demand forecasting. MAPE measures the average percentage difference between the predicted and actual values. It provides a relative measure of the forecast error, allowing for comparison across different datasets or forecasting methods.

Here ${x}_{t}$ is the observed values and ${\widehat{x}}_{t}$ are the estimated or predicted values An error has been defined as the difference between the actual and fitted values. The expressions for these KPIs are expressed below respectively;

9 $${\varvec{M}}{\varvec{A}}{\varvec{E}}=\frac{1}{{\varvec{N}}}\sum_{{\varvec{t}}=1}^{{\varvec{N}}}\left|{{\varvec{x}}}_{{\varvec{t}}}-{\widehat{{\varvec{x}}}}_{{\varvec{t}}}\right|$$

10 $${\varvec{R}}{\varvec{M}}{\varvec{S}}{\varvec{E}}=\sqrt{\frac{1}{{\varvec{N}}}\sum_{{\varvec{t}}=1}^{{\varvec{N}}}{\left({{\varvec{x}}}_{{\varvec{t}}}-{\widehat{{\varvec{x}}}}_{{\varvec{t}}}\right)}^{2}}$$

11 $${\varvec{M}}{\varvec{A}}{\varvec{P}}{\varvec{E}}=\frac{1}{{\varvec{N}}}\sum_{{\varvec{t}}=1}^{{\varvec{N}}}\left|\frac{{{\varvec{x}}}_{{\varvec{t}}}-{\widehat{{\varvec{x}}}}_{{\varvec{t}}}}{{{\varvec{x}}}_{{\varvec{t}}}}\right|\boldsymbol{*}100$$

## Results and discussion

Our analysis begins with a descriptive analysis of the performance indicators of players. In terms of winning probabilities, Roger Federer had a higher probability of winning compared to Novak Djokovic and Rafael Nadal. Novak had a lower standard deviation of winning probabilities than Rafael and Roger, indicating more consistent performance. Novak also had a lower coefficient of variation and interquartile range, further demonstrating his consistency.

Looking at aces, Roger had the highest number of ace points with 580, surpassing Novak and Rafael. Rafael had a lower standard deviation in the number of aces compared to the other two. Novak's minimum and maximum aces were 26 in 2004 and 518 in 2007, respectively. Rafael's ranged from 57 in 2004 to 310 in 2010, while Roger's ranged from 66 in 2020 to 695 in 2008. Examining double faults, Novak had a higher average number of double faults compared to the other players, although Rafael had a lower standard deviation in this aspect. Novak's minimum and maximum double faults were 21 in 2004 and 282 in 2010, respectively. Rafael's ranged from 59 in 2012 to 166 in 2015, while Roger's ranged from 11 in 2021 to 156 in 2004. When considering game dominance, Roger had a higher average game dominance with a mean of 2.685, compared to Novak and Rafael. Novak's minimum and maximum game dominance was 0.79 in 2004 and 3.27 in 2015, respectively. Rafael's ranged from 1.31 in 2004 to 3.76 in 2019, while Roger's ranged from 1.38 in 2021 to 3.61 in 2004.

**Fig. 3:**
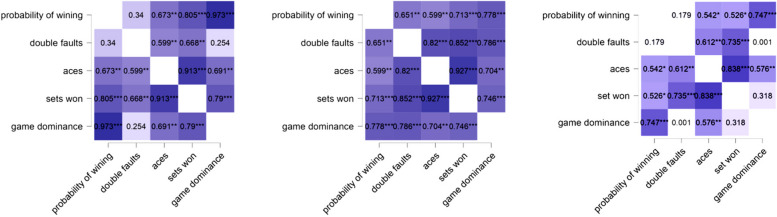
Pearson's r heatmap of Novak, Roger, and Rafael's key performance measures

Figure 3 presents a heatmap illustrating the correlation between performance measures and winning probabilities for all three tennis players. Analyzing Novak's statistical data, we observed a significant correlation between the probability of winning and aces, sets won, and game dominance. The strongest correlation was found between winning probability and game dominance (r = 0.973***), while the weakest correlation was observed between winning probability and double faults (r = 0.34).

Regarding Roger's career, we observed a significant association between winning probability and several factors, including double faults, aces, sets won, and game dominance. The strongest positive correlation with winning probability was found with game dominance (r = 0.778***), while the weakest correlation was observed with aces (r = 0.599***). Analyzing Roger's statistical information, we found significant correlations between winning probability and aces, sets won, and game dominance. Notably, we observed a strong positive correlation between winning probability and game dominance (r = 0.746***), while the weakest strong positive correlation was noted with double faults (r = 0.179).

## Comparison of Linear Time series and NNAR model

We process the analysis by checking the presence of stationarity in the series. To achieve this end, we apply the Augmented-Dicky fuller test on the data series. This test is useful in tracing the non-stationarity component from the data. If the p-value which results is less than the level of significance alpha = 0.05, the null hypothesis is rejected and concludes that the series is not stationary. The nonstationary series can be made stationary by applying a differencing or any other transformation method that aligns with the data characteristics. Initially, the series exhibited non-stationarity at level 0, so we applied the first differencing to achieve stationarity. Subsequently, all the series became stationary after the first differencing. Once the series became stationary, we proceeded to search for the most suitable candidate models. To accomplish this, we constructed a correlogram of the differenced series. From the analysis of the correlogram, it is found that the suitable ARIMA model for the series is ARIMA(2,1,3) as the lags of ACF and PACF are out of the boundaries on 2 and 3 lag.

For the application of the NNAR model, we proceeded as follows;

(I) first, the Box–Cox transformation was applied before estimating the model.
(II) secondly, the optimum number of non-seasonal lags p was identified for AR ($P$) process then the P lag was set to 1 and the optimal number of neurons identified was estimated by the formula; ${\varvec{k}}=\frac{{\varvec{p}}+{\varvec{P}}+1}{2}$. Here ${\varvec{p}}$ = 8 and ${\varvec{P}}$ = 1, where p shows the embedding dimension for non-seasonal time series. 8 non-seasonal lags have been used as input nodes [26]. In our model, there are 4 hidden layers. Practically speaking, hidden nodes are half of the input nodes. The function nnetar() has been utilized to apply a non-linear autoregressive technique for forecasting purposes on the performance indicators of each player. This function belongs to the forecast package for R and is capable of fitting a neural network model to a time series using lagged values of the time series as inputs.

The NNAR model showed the lowest values of RMSE, MAE, and MAPE among all models in predicting the performance of tennis players as noted in Table 2. For the performance indicators of NOVAK DJOKOVIC, the NNAR model for the probability of winning model value of RMSE = 0.01601, MAE = 0.01255, and MAPE = 2.1618 which is the least among all the models. Predicting the performance measure double defaults, for the NNAR model, RMSE = 53.0077, MAE = 41.7159, and MAPE = 35.3696 showing the least values among all the models applied. NNAR model for predicting the performance indicator, ACES, the RMSE = 104.2740, MAE = 85.51023, and MAPE = 28.7619. Similarly NNAR model for modeling the dominance performance indicator, the RMSE = 0.3597, MAE = 0.2989, and MAPE = 13.9719 which are the least among all the models.

As noted in Table 1, considering the modeling of the performance of Rafael Nadal, the NNAR model outperformed all other models in modeling his performance indicators. For modeling the probability of winning, the RMSE of NNAR is 0.0461, MAE = 0.04139, and MAPE = 4.9641 which is the least among all selected models Modeling the double defaults of the players, NNAR also showed the lowest values of RMSE = 20.5791, MAE = 14.8302, and MAPE = 15.8998. For ACES, the RMSE of NNAR is 60.195, 49.050, and MAPE = 27.5613. In modeling the dominance of the player from 2004 to 2022, NNAR also showed the lowest values of RMSE i.e. 0.5080, MAE = 0.3964, and MAPE = 16.1695. In the case of Roger Federer, the RMSE of the NNAR model for modeling the probability of winning is 0.0544, MAE = 0.0398, and MAPE = 4.9312. for double defaults, RMSE for the NNAR model is 35.9006, MAE is 26.1837 and MAPE is 75.8978 lowest among all the selected models. The second best model among the models is TBATS having RMSE, MAE, and MAPE lower than other models except for NNAR. For ACES from the year 2004 to 2022, the NNAR model showed the RMSE = 145.9614, MAE = 110, 6277, and MAPE = 57.69298 showed the least KPI values among all selected models. The second best model for modeling the ACES is TBATS and the model which showed the highest values of KPIs is ARIMA. NNAR model for dominance proposed the least values of RMSE, MAE, and MAPE with the respective values 0.4849, 0.3552, and 15.3458. It can be compared from the results of all performance measures that NNAR performed well as compared to ARIMA, ETS, and TBATS models for all three tennis players.

**Table 1:** Diagnostic measures of models predicting the performance of the tennis players

NOVAK DJOKOVIC			
**Probability of winning**	**RMSE**	**MAE**	**MAPE**
ARIMA	0.02126	0.01616	2.7436
ETS	0.02126	0.01614	2.7383
NNAR	0.01601	0.01255	2.1618
TBATS	0.02146	0.01675	2.9503
**Double defaults**			
ARIMA	56.5542	43.7351	57.2306
ETS	66.9577	49.8517	43.0383
NNAR	53.0077	41.7159	35.3696
TBATS	62.6287	45.3745	62.4586
**ACES**			
ARIMA	128.4564	109.9007	89.2184
ETS	144.0134	119.6025	106.7189
NNAR	104.2740	85.51023	28.7619
TBATS	144.3174	121.1243	102.2265
**Dominance**			
ARIMA	0.4705	0.3837	17.2226
ETS	0.3647	0.2891	13.8187
NNAR	0.3597	0.2989	13.9716
TBATS	0.4276	0.3493	20.3316
**RAFAEL NADAL**			
**Probability of winning**			
ARIMA	0.0660	0.04760	6.0412
ETS	0.0660	0.04760	6.0416
NNAR	0.0461	0.04139	4.9641
TBATS	0.0660	0.04768	6.0075
**Double defaults**			
ARIMA	24.6661	19.6842	20.9383
ETS	24.6674	19.6845	20.9398
NNAR	20.5791	14.8302	15.8998
TBATS	24.5742	19.4389	20.8931
**ACES**			
ARIMA	69.9294	59.6066	41.1588
ETS	69.9329	59.6110	41.1625
NNAR	60.1918	49.4050	27.5613
TBATS	69.5792	59.0015	40.9155
**Dominance**			
ARIMA	0.5712	0.4684	20.9455
ETS	0.5712	0.4684	20.9464
NNAR	0.5080	0.3964	16.1695
TBATS	0.5736	0.4608	20.0164
**ROGER FEDERER**			
**Probability of winning**			
ARIMA	0.0686	0.0509	6.1968
ETS	0.0686	0.0510	6.1972
NNAR	0.0544	0.0398	4.9312
TBATS	0.0699	0.0537	6.5880
**Double defaults**			
ARIMA	40.1647	29.6454	98.8794
ETS	40.1667	29.6439	98.8862
NNAR	35.9006	26.1837	75.8978
TBATS	36.5587	29.1475	94.0206
**ACES**			
ARIMA	202.3148	145.5033	60.13508
ETS	168.8342	116.2379	66.44011
NNAR	145.9614	110.6277	57.69298
TBATS	148.8688	118.6755	59.20205
**Dominance**			
ARIMA	0.5654	0.4243	18.4607
ETS	0.5655	0.4243	18.4622
NNAR	0.4849	0.3552	16.3458
TBATS	0.5672	0.4247	18.9381

**Fig. 4:**
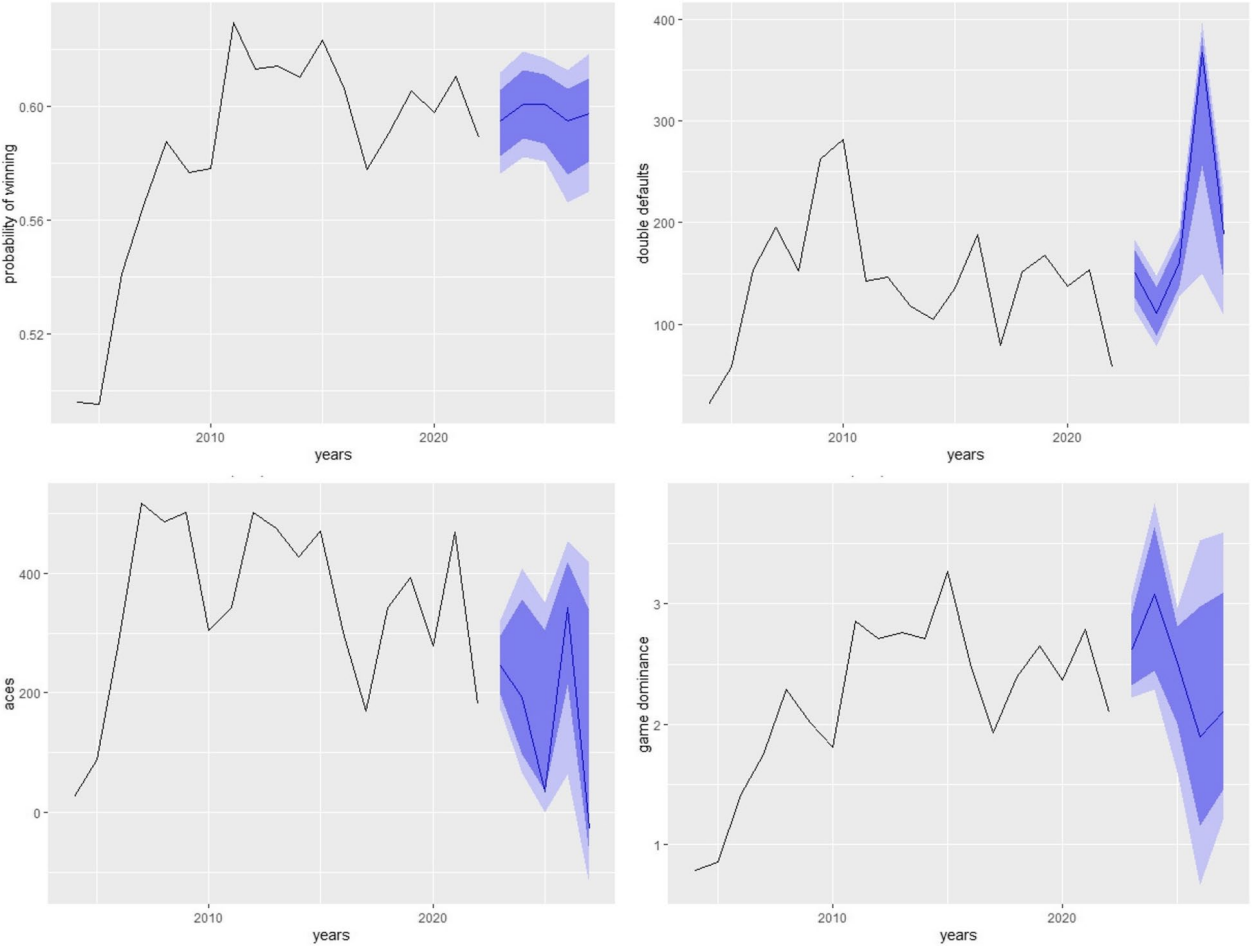
Short-term forecasting by NNAR (8,4) of performances measures of NOVAK

**Fig. 5:**
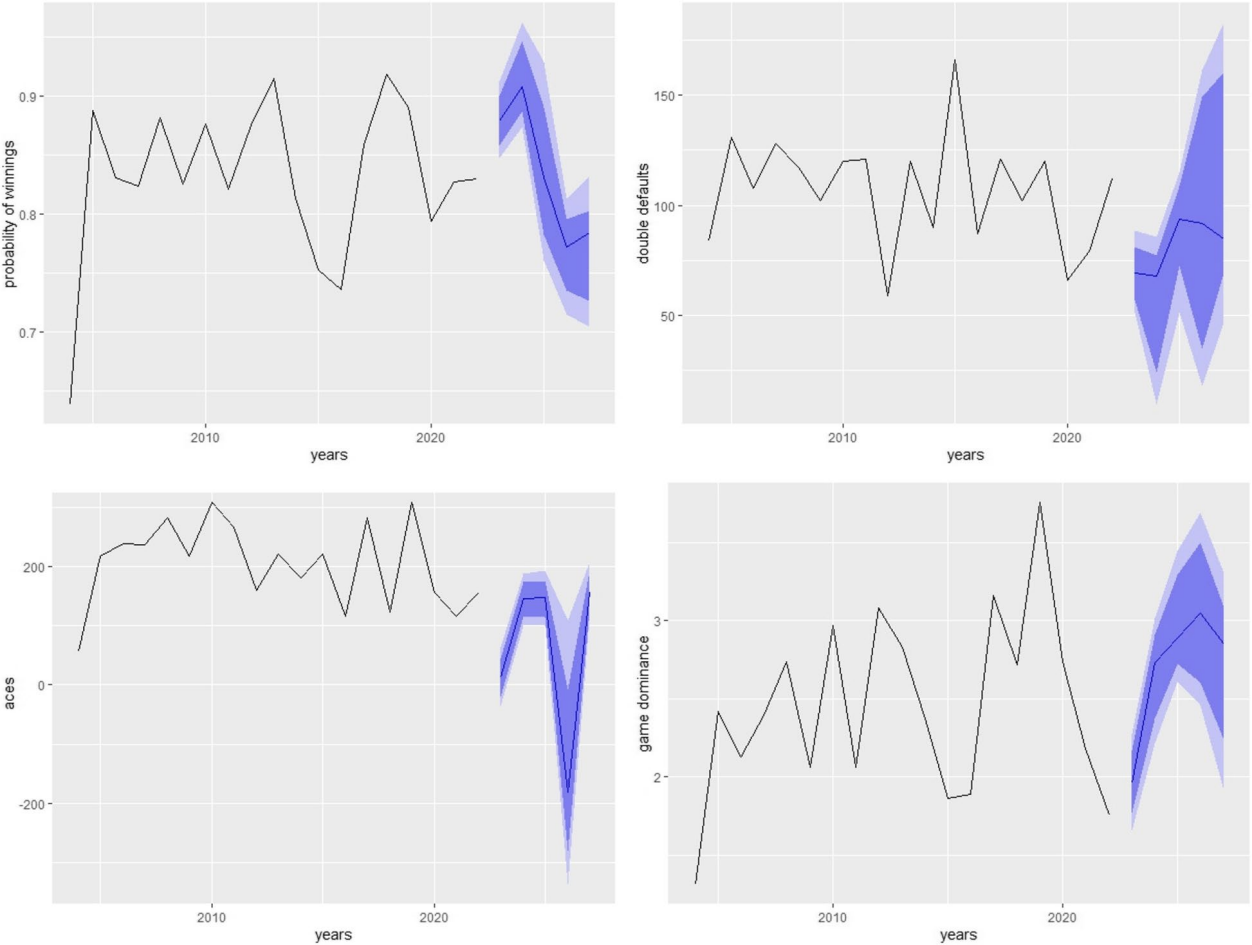
Short-term forecasting by NNAR (8,4) of performances measures of Rafael

**Fig. 6:**
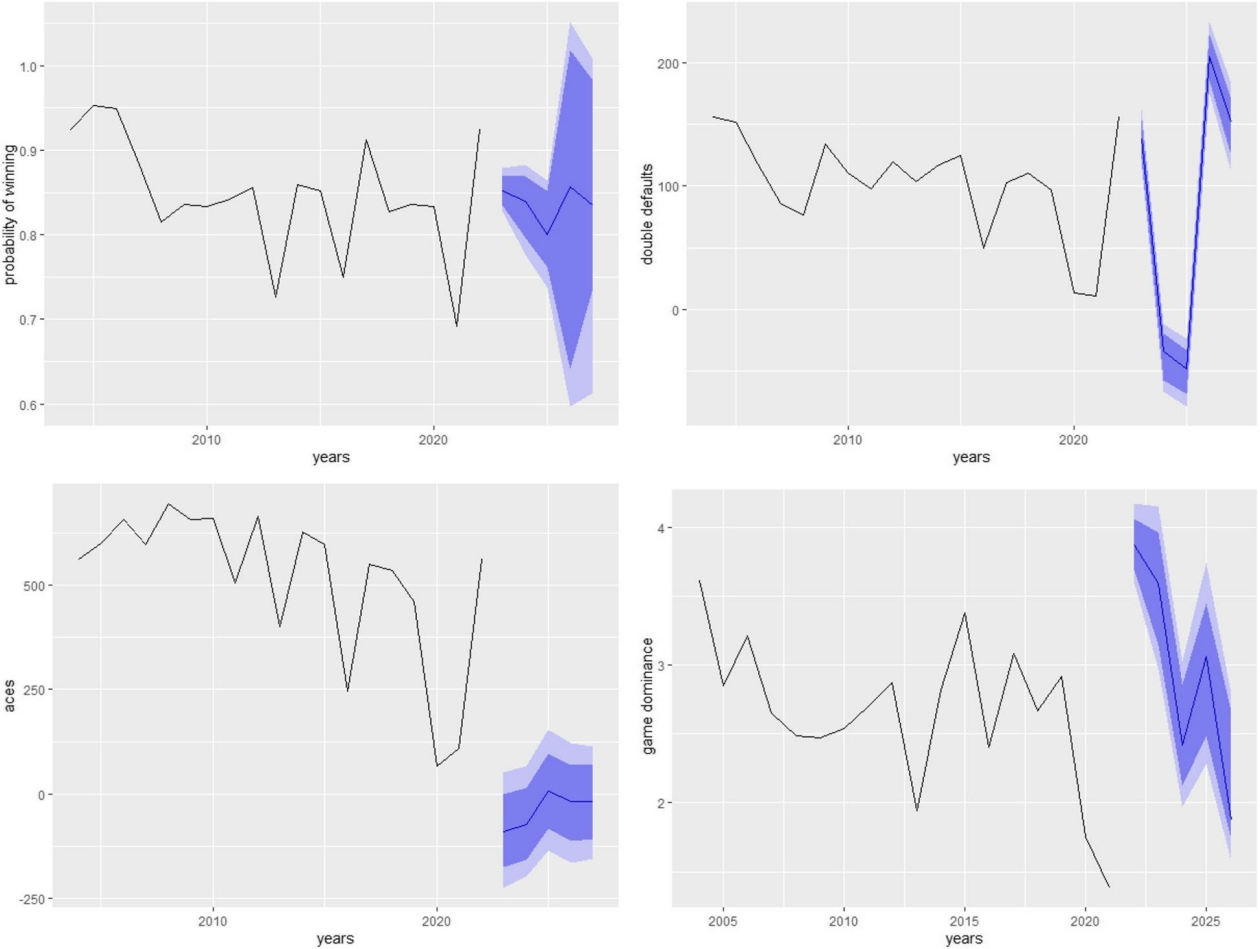
Short-term forecasting by NNAR (8,4) of performances measures of Roger

Figures 4, 5 and 6 shows the forecasting of different performance measures used in tennis for all three players using NNAR (8,4). NNAR outperformed all other models in modeling and forecasting purposes as it showed the lowest values of KPIs among all other models.

Considering the 5 years ahead forecasting from Table 2 it can be noticed that the probability of winning for Novak, Rafael, and Roger if they continue to play, will be 0.610, 0.7937, and 0.919 respectively. It can also be concluded from the study that aces of Novak, Rafael, and Roger will see ups and downs from 2023 to 2027 and will be 419.658, 218.735, and 24.317 respectively till 2027. Considering the game defaults, Novak will make around 78.933, Rafael will make 143.308 and Roger will be making 138.17 game defaults around 2027 and they also observed gradual ups and downs. The game dominance for Novak will see a constant pattern till 2027 and will remain around 2.628 if he continues to play till 2027 Rafael will observe a downward trend in game dominance to around 2.237 and Roger will be expecting to see an increase in the game dominance around 3.432 if he continues to play his game till 2027.

**Table 2:** Five years forecasting of performance indicators by NNAR (8,4) for all three players

NOVAK	Probability of winning with 95% C.I	ACES with 95% C.I	Game defaults with 95% C.I	Game dominance with 95% C.I
2023	0.638 **(0.6381,0.63828)**	18.024**(15.06,19.84)**	261.701**(261.63,261.77)**	2.555**(2.53,2.57)**
2024	0.586**(0.5860,0.5862)**	317.960**(316.93,319.18)**	51.193**(51.10,51.28)**	2.282**(2.26,2.29)**
2025	0.613**(0.6134,0.6136)**	301.812**(300.69,303.04)**	171.886**(171.80,171.97)**	2.600**(2.58,2.62)**
2026	0.581**(0.5810,0.5812)**	93.492**(92.26,94.79)**	149.504**(149.41,149.59)**	2.072**(2.05,2.09)**
2027	0.610**(0.6100,0.6102)**	419.658**(418.29,421.05)**	78.933**(78.84,79.017)**	2.628**(2.60,2.64)**
**RAFAEL**				
2023	0.8776**(0.8775,0.8776)**	191.930**(190.97,192.91)**	170.155**(170.13,170.17)**	1.854**(1.853,1.855)**
2024	0.8793**(0.8793,0.87944)**	144.734**(143.77,145,71)**	95.347**(95.323,95.370)**	3.149**(3.148,3.150)**
2025	0.7948**(0.7947,0.7949)**	187.187**(185.93,188.58)**	125.854**(125.82,125.86)**	3.058**(3.058,3.059)**
2026	0.7863**(0.7864,0.7864)**	178.562**(175.79,181.12)**	80.857**(80.83,80.88)**	3.828**(3.827,3.828)**
2027	0.7937**(0.7937,0.7938)**	218.735**(211.37,227.15)**	143.308**(143.28,143.33)**	2.237**(2.236,2.237)**
**ROGER**				
2023	0.8362**(0.8361,0.8363)**	45.97**(36.90,54.86)**	121.252**(120.69,120.83)**	2.557**(2.540,2.562)**
2024	0.740**(0.7405,0.7407)**	68.44**(59.48,76.51)**	62.069**(62.47512,63.62)**	2.552**(2.543,2.560)**
2025	0.8901**(0.8900,0.8901)**	48.37**(39.35,57.97)**	54.192**(53.788,55.00)**	2.729**(2.712,2.748)**
2026	0.7724**(0.7723,0.7725)**	8.44**(10.29,17.36)**	145.701 **(145.22,146.15)**	3.329**(3.312,3.345)**
2027	0.9190**(0.9193,0.9194)**	24.317**(14.26,33.69)**	138.17**(137.73,138.65)**	3.432**(3.417,3.443)**

## Conclusion

Previous studies [9, 16, 22–25] on forecasting match results in tennis have commonly relied on official rankings to infer the probability of a player winning a match. However, the reliability of these rankings-based models has been questioned. In our study, we took a different approach by utilizing various time series models that leverage the historical performance indicators of individual players, which in turn contribute to their official rankings in ATP. To assess the performance indicators of players, we considered key factors such as the probability of winning, aces, game defaults, and game dominance. These indicators are recognized as important measures in determining player rankings. Consequently, we evaluated different stochastic linear and non-linear time series models to effectively model and predict these performance indicators. Based on our analysis, the neural network auto-regressive model NNAR (8,4) outperformed all other selected models in terms of model selection criteria, namely RMSE, MAE, and MAPE. As a result, this model was employed for the purpose of forecasting. Short-term forecasting was performed up to 5 years ahead, assuming players would continue to participate in the game. This study emphasizes that relying solely on the probability of a player winning a match may not accurately reflect their performance or ranking in ATP. Considering the performance measures which are time-dependent plays a significant role and should not be overlooked. Neglecting this factor can lead to misleading conclusions. Moreover, this study holds great potential for benefiting players by providing insights to enhance their performance indicators in the future. By analyzing the results and gaining valuable knowledge, players can make targeted improvements to their performance. It's important to note that this study serves as a case study, focusing on the performance of three players and comparing different time series models. Given the outcomes and insights derived from this research, we recommend the NNAR model as a suitable choice for predicting various indicators in the tennis game in the near future.

## Data Availability

The data is available online on https://www.ultimatetennisstatistics.com/playerProfile?playerId=4742&tab=timeline.
